# Usability of self-reported assessment of work functioning in municipal occupational rehabilitation teams: A qualitative study

**DOI:** 10.3389/fresc.2022.971574

**Published:** 2023-01-19

**Authors:** Anne-Mette Hedeager Momsen, Merete Tonnesen, Birgitte Zwicky-Hauschild, Claus Vinther Nielsen, Reuben Escorpizo, Vivian Langagergaard, Christina Malmose Stapelfeldt

**Affiliations:** ^1^ DEFACTUM—Public Health and Rehabilitation Research, Central Denmark Region, Aarhus C, Denmark; ^2^ Department of Clinical Social Medicine and Rehabilitation, Gødstrup Hospital, Herning, Denmark; ^3^ Department of Public Health, Aarhus University, Aarhus, Denmark; ^4^ Department of Rehabilitation and Movement Science, The University of Vermont, Burlington, VT, United States; ^5^ Swiss Paraplegic Research, Nottwil, Switzerland; ^6^ The Danish Clinical Registries (RKKP), Aarhus N, Denmark

**Keywords:** return to work, sick leave, social workers, focus groups, surveys and questionnaires

## Abstract

**Aims:**

This study aimed to explore (1) whether self-reported assessment on work-related functioning, workability, return-to-work (RTW) self-efficacy, and expectation was useful in the professionals’ assessment of sick-listed workers and could guide referral to interventions and (2) whether self-reporting in addition to “usual practice” could improve the RTW dialog and involvement in case management.

**Methods:**

The qualitative study took place in two municipal job centers in 2021. The assessment was based on the Work Rehabilitation Questionnaire, RTW-Self-efficacy Scale-19, and single items of self-rated health, workability, and RTW expectations. Sick-listed workers (*n* = 36) were interviewed by telephone. Three focus-group interviews were conducted with professionals who had used the questionnaire. Data were coded and analyzed thematically.

**Results:**

Three themes with seven subthemes emerged: (1) accessibility; (2) one tool in the RTW toolbox (subthemes: a supplementary tool, a tool for reflection, facilitating interdisciplinary communication, and enhancing active participation); and (3) the value of “ticking boxes” (subthemes: good days, bad days, the issue of power, and the cultural meaning of words).

**Conclusion:**

The professionals would not recommend the present questionnaire for use during their rehabilitation team meeting for assessment, interdisciplinary communication, or choice of interventions. However, using the parts assessing RTW self-efficacy and expectation combined with a dialog may be of value early in the RTW process. The self-reporting assessment tool was perceived to be meaningful to some sick-listed workers, as it provided reflections on important aspects of the RTW process. Some workers believed that it might contribute to the rehabilitation team, and thus, it could improve their involvement.

## Introduction

The prevalence of people with disabilities who experience health and occupational disparities varies due to disability measures and survey methodologies (1). Long-term sickness absence is a significant public health problem and may lead to permanent labor market exclusion (2). Therefore, occupational rehabilitation (OR) aiming at inclusion and return to work (RTW) is advantageous for individuals and society (3). The role of the Danish municipal job centers is to provide OR to improve work participation among working-age persons. Sick leave may be the start of a long assessment process of workability, medical examinations, care, and tryouts. The Danish social welfare legislation places great importance on the primacy of work. Reforms in 2013–2015 aimed at reducing disability pensions, representing a welfare discourse explicitly linking benefits to requirements regarding work participation (4, 5). Along with the reforms, multidisciplinary municipal rehabilitation teams (RTs) were introduced; the RT meetings include the person on sick leave, professionals from the municipal job center (appointed as case managers), health and social departments, and a regional healthcare coordinator (HCC) (a physician with expertise in clinical social medicine) (6). The RT establishes which OR interventions are needed to enable the RTW process.

**Table 1:** Characteristics of the sick-listed individuals (*N* = 52) testing the questionnaire.

Gender, *n* (%)	
Female	40 (77)
Male	12 (23)
Age, mean (min–max)	40.5 (20–60)
Education, *n* (%)	
None	13 (25)
1–2 years	8 (15)
3 years	13 (25)
>3 years	18 (35)
General health, *n* (%)	
Excellent	0 (0)
Very good	3 (6)
Good	8 (15)
Less good	26 (50)
Poor	15 (29)
Workability, *n* (%)	
0–3	36 (69)
4–7	13 (25)
8–10	3 (6)
RTW expectations, *n* (%)	
0–3	13 (25)
4–7	19 (37)
8–10	20 (38)
Herning municipality	
Sick leave duration, *n* (%)	
>22–104 weeks	14 (54)
>104–208 weeks	6 (23)
>208 weeks	6 (23)
Ringkøbing-Skjern municipality	
Sick leave duration, *n* (%)	
>22–104 weeks	12 (52)
>104–208 weeks	8 (35)
>208 weeks	3 (13)
Missing	3

RTW, return-to-work.

Assessment and expenses for work-enhancing interventions constitute up to 10% of the municipalities’ annual budget (7, 8). According to the Danish Sickness Benefit Act, the job centers are obliged to initiate OR for adults on sick leave benefits after 8 weeks; benefits are based on workability; therefore, assessment is crucial. The usual practice has been to collect medical information from general practitioners (GPs) and specialized medical departments. Furthermore, the sick-listed person is asked to fill in a document in cooperation with the case manager before the RT meeting (**Supplementary Appendix**); the quality varies and may be inadequate regarding which interventions are needed. Moreover, the evaluation of the reforms has revealed a need for a more systematic assessment of workability to assure which interventions to initiate (9–11). Furthermore, an instrument to align practices across municipalities in case management is lacking.

Professionals often fail to predict the duration of sick leave, whereas self-rated workability seems to be more accurate (12).

The International Classification of Functioning, Disability and Health (ICF) was approved by the World Health Assembly in 2001 as a framework covering bodily, social, and participatory aspects of functioning (13). Thus, the ICF captures a comprehensive view of disability relevant to rehabilitation. An ICF core set for OR was developed on which the “Work Rehabilitation Questionnaire” was based (14, 15).

A study on RTW facilitators revealed several modifiable areas, e.g., dialog, to increase awareness of limits and strengths. Communication processes may enhance RTW; thus, the use of dialog may increase the cooperation between the sick-listed person and the case manager on goal setting and OR initiation (16). Furthermore, a review revealed a gap between the knowledge of the impact of personal factors and actual assessment within OR (17).

Personal factors, e.g., self-efficacy, are of importance regarding RTW (18, 19). Self-efficacy can be defined as “a personal judgment on how well one can execute courses of action required to deal with prospective situations” (20). It is demonstrated to be strongly related to work participation, e.g., job seeking and other behavioral outcomes (21). Assessment and dialog may increase the sick-listed workers’ awareness of their limits and attitudes (16); however, RTW self-efficacy is not used in usual case management. The present study included a systematic assessment of job-related self-efficacy, workability, and health, the measures that have proven useful in OR (22).

### Purpose

The aims were to explore (1) whether self-reported assessment on work-related functioning, workability, RTW self-efficacy, and expectation was useful in the professionals’ assessment of sick-listed workers and could guide referral to interventions and (2) whether self-reporting in addition to “usual practice” could improve the RTW dialog and involvement in case management.

## Methods and materials

The research questions were as follows:

How do the professionals experience the use of the added self-reported information from the sick-listed workers in preparation to the RT meeting?

Does it facilitate interdisciplinary communication and choice of intervention?

Does the use of a self-reported assessment stimulate the sick-listed worker to participate in the RTW process; does it increase the experience of involvement?

### Design

A qualitative method was applied with individual telephone interviews and focus-group interviews to enable in-depth reflections about informants’ experiences of using the questionnaire. The questionnaire data were used to characterize the group of sick-listed workers. Telephone interviews were chosen (to follow physical distancing regulations during the Covid-19 epidemic).

Focus-group interviews were conducted with municipal professionals and HCCs (RT members). This method allows the exchange of diverse experiences and views.

### Settings and eligibility criteria

The interviews took place in two municipal job centers in Central Denmark Region, serving 90,000 and 57,000 people, respectively, with OR services, elder care, schools, etc.

Recruitment of participants took place from February to October 2021.

First, the inclusion criteria were sick-listed persons referred by the RT to a resource-building program. However, as these referrals declined in 2021, persons referred to job training were included. All persons belonging to these groups were eligible for inclusion.

### Sick-listed workers

In total, 52 answered the questionnaire, of whom 6 received help from a social worker to answer. The majority of the participants were women (77%), of whom 79% rated their general health as “less good” or “poor.”

Among the responders, 36 were interviewed. Seven were unreachable or did not respond, and nine declined. They primarily provided reflections on answering the questionnaire, as none had yet participated in RT meetings, which were taking place during the last months, followed by referral to interventions [24 were granted job training, 5 with modified jobs (reduced-hour jobs), 6 with resource-building programs (one municipal only), and 2 with disability pension].

### Professionals

Three focus-group interviews were conducted with 14 professionals experienced in using self-reported data (in 1–15 cases). The RT comprised an RT coordinator and an HCC; in one municipality, social workers from diverse municipal departments participated, whereas in the second municipality, only case managers from the job center were interviewed.

### Materials

The self-reported assessment (35 items) was a combination of the Work Rehabilitation Questionnaire (WORQ) (14, 23) and Return-to-Work Self-efficacy Scale-19 (RTW-SE 19) (24, 25), single items on self-rated health from SF-12, RTW-expectation from Örebro Musculoskeletal Pain Screening Questionnaire (QMPSQ-DK version 2.0) (26), and a single item on workability from the Work Ability Index (WAI) (27, 28).

WORQ was developed in 2010 based on the ICF Core set for vocational Rehabilitation (14) with categories from the ICF (29). WORQ can be used to describe work-related functioning, goal setting, and monitoring. It is free to use and has been cross-culturally adapted to 13 languages (30), including Danish, and validated across populations (18, 31–34). Correlations were found with related measures, e.g., SF-36 and EuroQoL (14, 23, 35). After the 32-item version (32), a 13-item version was developed, showing good internal consistency (Cronbach's alpha 0.96), test–retest reliability (intraclass correlation 0.91) (35), and convergent validity with EuroQoL (*r* = −0.65) (31). The 13-item brief version was used in this study.

The RTW-SE 19 was cross-culturally adapted to Danish (36) and has shown good reliability, validity, and responsiveness across diagnostic groups (36–38). The total self-efficacy score (0–190) is a strong RTW predictor among long-term sick-listed persons (18), cancer survivors (39), and persons with mental disorders (37). A review confirmed consistent positive associations with positive RTW outcomes (40).

One item on general health, “In general, would you say your health is (excellent, very good, good, fair, poor),” has proven to predict RTW (19, 41).

Workability is a generic term including all aspects, “Assume that your work ability at its best has a value of 10 points. How many points would you give your current work ability?” (0–10), and is derived from the WAI (27). WAI has been translated into 24 languages, has been used in practice and research for decades (28), and has been proven to predict RTW (42).

One item of QMPSQ-DK, “In your estimation, what are the chances that you will be able to work in six months?” (0–10), was also used. Full QMPSQ-DK version 2.0 has proven to predict RTW among musculoskeletal injury and low back pain populations (26).

### Usual case management practice

**Figure 1:**
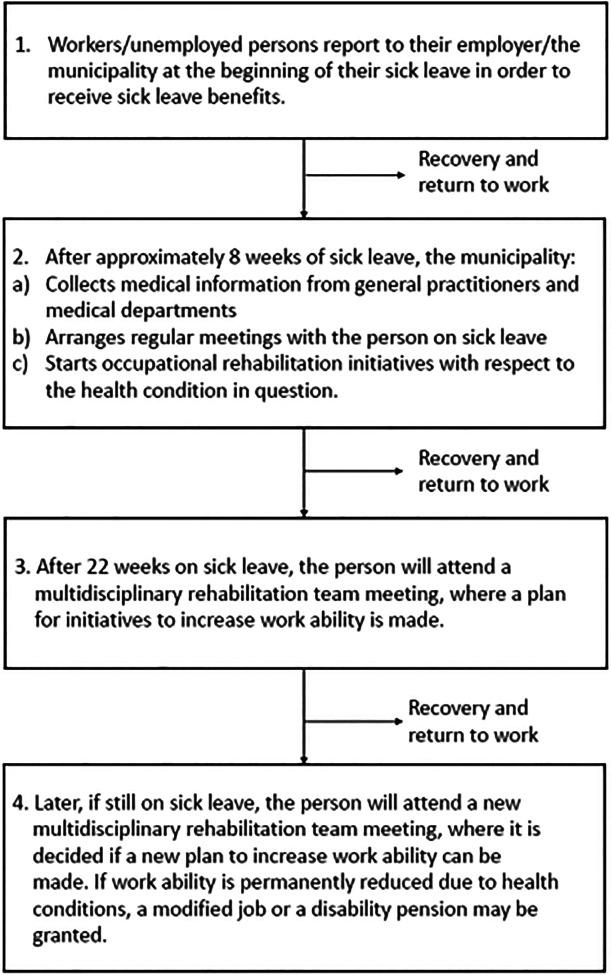
Occupational rehabilitation for persons on sick leave benefits in Danish municipalities.

The job centers are responsible for OR in the municipalities, granting benefits to workers/unemployed persons according to their entitlement, health, and workabilities (Figure 1). The case manager (social worker or administrative employee) is responsible for preparing the case before the RT meeting, gathering the information needed (the sick-listed worker’s curriculum vitae, professional skills, health status, social network, and work-related goals) to enable the RT to suggest an OR plan (**Supplementary Appendix**). Consequently, the case manager must make a summary of the individual’s situation.

All RT members are required to read the summary, medical information, prior attempts of job training, rehabilitation, and treatment before they meet to discuss a plan and coordinate further actions. The sick-listed person participates in the meeting, most often accompanied by a relative and the case manager.

The case management and RT practice differ between municipalities, e.g., regarding the diversity of professionals and municipal departments in meetings and the summary's scope and details.

### Introduction of a new practice

An information letter, including a link (Survey Exact access), was sent to the sick-listed worker's private email. The questionnaire could be answered before or at the individual meeting with assistance from the case manager.

The case managers were instructed on how to use the information for a dialog in addition to usual practice, and the study's aim to qualify their assessment was underpinned. They were responsible for follow-up on the answers and sending them to the RT members with the usual information.

An introduction to new participating case managers took place, as the usual 22 weeks of sick leave were prolonged three times up to July 1, 2021 due to Covid-19, and therefore, the RT meetings were postponed.

### Interviews

Two weeks after answering the questionnaire, telephone interviews were conducted by an experienced social worker (JBK) and a research assistant. An interview guide was developed, exploring opinions and experiences of the self-administered questionnaire. On average, interviews lasted 20 min. Questions included practical issues, e.g., accessibility, usability of the questionnaire in regard to both their RTW process and collaboration with the case manager, and the like. Furthermore, they were asked whether they believed the answers were used by the RT.

For focus-group interviews, the case managers and HCCs with experience in using questionnaire-based information from at least two RT meetings were invited. In one municipality, all participants were from the job center, whereas social workers from other departments (children and family, disability and psychiatry, healthcare center) participated in the other municipality's RT meeting.

The focus-group interviews were led by an anthropologist (MT) and an experienced social worker (JBK). An interview guide was developed and served as a checklist to make sure the same questions were asked but with room to pursue themes of interest that arose during the discussion. Interviews started with open questions on their experiences with the questionnaire-based information. The following questions included usability in connection with dialog and for the RT meeting: whether answers contributed to preparation for the RT meeting, for instance, interdisciplinary communication, and to qualify choice of interventions.

### Analysis

All interviews were audio-recorded and transcribed. Transcriptions were read thoroughly twice (MT and A-MHM); codes were generated, then discussed within the author group, and finally refined (43). NVivo 11.0 QSR software was used to manage data. The data analysis followed an iterative process. A preliminary thematic analysis (44) was made and then discussed with an interdisciplinary group (authors JBK and KV). Data were revisited, and themes were adjusted and refined. Findings are illustrated with quotes from informants.

### Ethics statement

The research was conducted in accordance with the declaration of Helsinki (45). The study did not need approval by the Medical Ethical Committee of the Central Denmark Region. Informed consent was obtained, and potential participants were contacted by one of the interviewers and presented the study purpose and data management. The participants were anonymized. In a few cases, when distraught about their situation, the interviewers gave room to explain their situation. Participants in the focus-group interviews were informed at meetings held in the municipalities.

## Results

In our analysis of the interviews, three themes with a total of seven subthemes emerged: (1) accessibility; (2) one tool in the RTW toolbox (subthemes: a supplementary tool, a tool for reflection, facilitating interdisciplinary communication, and enhancing active participation); and (3) the value of “ticking boxes” (subthemes: good days, bad days, the issue of power, and the cultural meaning of words).

### Accessibility

During interviews, the case managers explained that some sick-listed workers declined to answer the questionnaire after realizing it was not mandatory, some agreed to participate but gave up when they saw the questions, and a few called their case managers regarding specific items. These explanations made their colleagues voice their concerns: Were there too many questions? Did some wording require elaboration and explanation? Did the questionnaire require a certain level of reflection and understanding?

We therefore inquired whether the sick-listed workers found the questionnaire easy to access and understand. Thirty-two of the 36 had responded to the questionnaire online, and all found it easy to get and complete. Six received help from their social worker, spouse, or mentor. Several mentioned that they were used to questionnaires. Most found it to be user-friendly. “*This and all the other questionnaires are super easy to access; I don't have a computer, but it worked very well just using my phone …. the questions were easy to understand*” *(HM)*.

A few wished for more nuanced options for answers:

*“It was reasonably straightforward to fill in the questionnaire, even if I did miss a few more options; it was too much either or.” (JJ)*

Only a few found words difficult to understand or that questions lacked clarity.

### One tool in the RTW toolbox

When asked about their experiences, professionals and sick-listed workers offered different opinions. The majority, however, conveyed similar opinions to a statement proposed by a sick-listed worker: “*[the questionnaire] can be used as a tool, but not as a fact sheet*,” meaning to the professionals that it is merely one tool in the RTW toolbox to access information. Generally, the informants underscored that the questionnaire cannot stand alone, as it does not convey all the important aspects in need of illumination nor does it provide any contextual factors to explain the answers given. It therefore needs to be supplemented with a dialog. The interviews revealed how the questionnaire could be used for different purposes.

#### Professionals: a supplementary tool

Most case managers used the questionnaire before the RT meeting for dialog, asking follow-up questions to elaborate on the answers, to gain insight into the person's ideas for progress, e.g., “*what would it take for you to move from x to y?*”. Others used the questionnaire as inspiration to describe the case. Some sent the answers to the RT without a dialog. Finally, a few used it as a follow-up tool.

The professionals, beyond case managers, just skimmed the answers in preparation for the RT meeting. It came last in the string of documents, making it “*drown in other documents*.” They found that the answers could contribute to the RT meeting as a supplement. Generally, however, it yielded little new information but confirmed what had already been documented, as one case manager explained:

“*It is a fine supplement, I agree, but it does not reveal any surprising new to the case. With the many cases we have, there is a limit to how thoroughly we can go through a questionnaire like this. However, in 2–3 cases we could see that these were complex cases; the sick listed workers had little faith in a RTW and little trust towards their boss so we had to provide a mentor. [Generally, however] I doubt we do anything differently [concerning choice of interventions] because of this questionnaire.*”

Other professionals agreed that the questionnaire did not help in choosing interventions in any substantial way.

One had hoped the questionnaire would provide an overview: “*that we could equate it with descriptions, but clearly, we cannot*,” whereas another professional found that the questionnaire lacked a focus on resources and strengths, “*some of this is rather negative which I find a pity. Because in that situation maybe you need something else to make a mental move—I miss that. I don't see that in the questionnaire*.”

All professionals found that a dialog was needed to explore the answers provided in the questionnaire, yet they all agreed that the time allocation for an RT meeting allowed little time to go through the questionnaire and ask the sick-listed workers to elaborate on resources and strengths.

#### Sick-listed workers: a tool for reflection?

Two of three sick-listed workers said “no” when asked whether the questionnaire had helped them clarify how to RTW; it did not yield ideas or match their situation. However, some had used it to prepare for the RT meeting, indicating the themes likely to be discussed. It made them “*stop to reflect*” on their present and future situation, on work-related functioning and strategies, e.g., which information to give an employer and colleagues:

“*… how am I actually doing? And how could you have been doing? Filling in the answers may also make you approve of some of the choices you have made. … I made the right choice of accepting that I cannot perform like I used to*”. (GiSA)

“*I found rewarding the part about telling colleagues and the boss about how you feel, psychologically and physically, as that might help regarding the job tasks, they give you. You know, adapt the tasks to one's level of functioning*.”

While answering, a few realized that they were closer to/further from work than expected. Others found the questionnaire provided a quick overview which might contribute to a more efficient RT meeting.

Some did not find it useful for reflection, as those issues had already been discussed with professionals, e.g., psychologists. The irrelevance was linked to feeling insecure about their health situation and thus future work possibilities: “*I'd like to return to work but how and what the future brings … in that sense the work-related questions were difficult.*”

A resignation was expressed as no progression had happened for years: “*Why should a questionnaire change anything?*” Others expressed “*maybe*” or “*I don't know*,” as they were insecure about what an RT meeting entails; one said: “*It didn't provide me with any new reflections, but maybe the professionals will gain some information*.”

#### A tool to facilitate interdisciplinary communication?

The professionals found that the questionnaire did not make any difference in such regard. Here is a dialog between professionals:

“*In terms of our interdisciplinary communication, it [the questionnaire] hasn't made much change*.

*No, not for us, the rehabilitation team, but I do see the idea of using the questionnaire in the citizen/social worker collaboration.*

*Well, I also see the point of that, but only in a shortened and more specific version, and again with more dialogue, that is making more space for description. Because there are several fine questions in the questionnaire.”*

These considerations were repeated in all focus-group interviews. All suggested that the case managers should make a summary of the most important issues from the questionnaire in preparation for the RT meeting.

Some suggested that parts (RTW-SE-19) of the questionnaire could be a useful tool in the communication and collaboration between a sick-listed worker and a social worker or earlier in the RTW process when the sick-listed worker is closer to working life and have a specific job as a reference:

“*Sure. Maybe it [the RTW-SE19] is also useful in dialogue with the employer, as part of the round table dialogue that's offered the employer to participate in—in a dialogue of what we can do together.*

*I will consider using [the RTW part] when having a dialogue with the job consultant, I think that would work well.”*

Other professionals agreed, arguing that a question such as “how confident are you that you can suggest alterations at work to your boss” only makes sense if it can be directly related to a concrete workplace and boss and would not make sense if a person cannot return to their former job or had not been employed for some years. They would not expect a person to be able to imagine what things might be like in an unknown future job situation.

Several professionals also mentioned how the questionnaire could be used in follow-up dialogs.

*“… which interventions have been put in place, and what effect do they have? And regarding numbers it's easy to follow up—talking about “you say 6, what would it take to make it a 7?”*

#### A tool to enhance active participation?

The questionnaire made little difference regarding the active participation of the sick-listed in the RT meeting. A case manager said:

“*It would be so nice to have a tool that really brought in the voice of the sick-listed worker. In this format, the questionnaire does not do that*.”

The sick-listed workers somehow differed in opinion; they hoped that answering the questionnaire conveyed *their* voice and thus made their perspectives on their condition, abilities, and needs visible to the RT.

One sick-listed worker found it difficult that his level of functioning was not visible to others, at first sight, but

*“this questionnaire gives me a right – or how can I put it – to tell how I am”. (JB)*

A few emphasized that answers “*are MY words*”, thus valuing the possibility of answering questions with no interference by a professional: “*easier than to explain it all to a social worker*.”

### Value of ticking boxes?

All agreed that the questionnaire ought to be followed up by a dialog, and ticking gave little indication of functioning and needs.

#### Good days, bad days

Approximately one-third problematized; it only provided a snapshot but did not catch the experienced fluctuations in functioning.

“*I have some good days, when I can and will do things, and then I have some bad days, where I just cannot, and it is difficult to put into boxes*.”

The fluctuations in their condition made it difficult to estimate functioning on a scale.

“*… it couldn't describe all of it, because my bipolar condition goes up and down (…) It depends on which day you ask me, so I am not sure that it gives a real picture*.”

They underscored the need for dialog or the possibility of additional remarks.

Professionals agreed that they needed more description to ascertain the contextual factors; one said: “*What would have been interesting is whether there are changes compared to how it usually is*.”

#### The issue of power

Power of words and ticking interweaved reflections in both informant groups, the potential risk of being misunderstood or worrying that important decisions could be made by professionals on account of answers from a questionnaire. Some argued that it was a new world “*being in the system*”; they were unacquainted with procedures and insecure about the use of answers. Others had been in the system for a long time and felt little helped, hunted, and wary of what to say to whom. Some expressed that a questionnaire is not an innocent tool; it can potentially harm or misrepresent a case.

“*The hard part was—it's about your life, or how can I put it, you have to be careful what you write, you know, make sure you provide the whole picture. […] I was a bit afraid to make myself better than I am—I might have had a really good week, but the coming week is another story*.”

“*The question is how honest are you in assessing yourself? As a working tool it's fine, but it should not be used to say: ‘You wrote this – now we send you to work 15 hours a week’*.”

These quotes underscore a similar concern to the professionals that sick-listed workers would consider carefully how to fill in the questionnaire, fearing that it might harm their process and the decisions made if they answered too positively. A social worker explained how some persons might worry if the professionals at the RT meeting exercise their power to stop benefits. Professionals used words as “*fear, scared*” and “*stressed, pressurized*” to be pushed into a job situation where one might fail.

#### Cultural meaning of words

While ticking boxes, the sick-listed worker decides how to interpret the words, as there is usually no dialog about the meaning. Some professionals aired concern about how words are interpreted very differently, e.g., “depressed” or “anxious.” They pondered cultural differences across Denmark, “Crisis and conflicts—that is not something we have here in this area [western part of Denmark],” “we don't talk about those issues in the open.” Discussing with colleagues made them think about their practice and which questions they ask. Some became curious to revisit the study respondents’ answers more carefully.

### Single items

General health was considered very relevant by professionals, although it would need more elaboration through a dialog; professionals pondered whether “health” would be considered physical rather than mental health.

Workability was considered difficult to answer in situations of long-term unemployment and due to the importance of context: “*Right now I am unable to work but maybe with treatment I might be able to work again … so what kind of job do you have in mind when you respond*.”

Whereas the RTW expectation item was generally considered very relevant: “*It could be great to ask this question just after starting sick leave. And ask again later, to see how the person answers over time—getting worse if there is no progress. Because I believe most people would respond, in the beginning of their sick leave, that they will return to work*.”

The focus groups also raised discussion concerning who should use the questionnaire and when. It became obvious there was little knowledge among professionals of what tools were used in municipal RTW management.

## Discussion

### Summation of findings

Generally, the professionals did not find the self-reported information useful to qualify the RT's assessment of functioning nor did it enhance interdisciplinary communication or qualify the choice of intervention. Thus, no recommendation is being made for an implementation in its present format for use at RT meetings. However, parts regarding RTW expectations and self-efficacy could be useful tools in the communication preferably earlier in the OR process. Lack of common understanding of use of a dialog-based tool and the need for more descriptive answers and summary provided to the RT were given as reasons.

However, the overall sentiment was that a self-reported assessment might be a relevant and useful tool combined with a dialog, applied at the beginning of a sick leave spell, or used repetitively.

Differences in the beliefs and perceptions of words among professionals concerning the correlation between daily life and work-related functioning are of importance (16).

Sick-listed workers’ perceptions of the usability varied; it could contribute to preparation for the RT meeting, and it fueled reflections on their RTW process. Enhancing involvement and incorporation of the individual's knowledge and experiences of concern to the process is important in the RTW trajectory (46).

### Power imbalance and lack of knowledge

The well-known dilemma of “control over clients against support to clients” was raised by both informant groups (46). Some experienced the questionnaire part of a power imbalance between the professionals and the persons on sick leave. Ticking boxes may not be perceived as an innocent act but may possibly impact one's future. Thus, words can be (mis)used and may influence the course of the RTW process including decisions about social security benefits. This professional decision-making power is an issue that remains to be considered when evaluating self-reported work ability in an RT setting.

Sick-listed workers expressed a lack of knowledge of OR practices and which support to expect from the RT. Individualized professional attitudes toward the information provided in each case and a general lack of evidence-based case management practice revealed that the assessment of functioning and workability is yet to be systematized and structured.

### Strengths and limitations

Research has emphasized the importance of multifaceted collaboration between various stakeholders in OR (47), but studies are scarce on how cooperation works in practice (12, 16). This study investigated the addition of a self-reported tool to the usual practice among professionals in Danish OR settings, and despite the negative recommendation, future studies could explore the use of RTW parts among sick-listed workers at an earlier stage in the RTW process.

The study coincided with Covid-19 lockdown, which prolonged recruitment significantly. Thus, the intervention might have lost fidelity between intent and implementation, as the time lapse between the introduction and interviews was considerably longer than initially planned. The professionals were keen to be involved but emphasized the effort it had taken to recruit sick-listed workers. Information might be lost in transition/passing on to colleagues; the aims of using the answers for a dialog may be forgotten.

The sick leave period was extended; therefore, informants had been on sick leave for much longer than in a non-Covid-19 reality. Another limitation might be related to recall difficulties; the time between the questionnaire response and telephone interview was up to 3 weeks.

### Implication for practice

Both informant groups were positive toward the RTW questions on workability, self-efficacy, and expectations. These constructs are found to be significant in RTW interventions (22, 48, 49), and early in the OR process, these could be relevant. Furthermore, a shorter questionnaire (e.g., 10-item RTW self-efficacy) is easier to use (38).

## Conclusion

The professionals would not recommend the questionnaire in its present format in RT meetings, neither for assessment of sick-listed workers functioning nor to qualify the choice of intervention. However, some of the questions were considered relevant and useful at an earlier stage of job centers’ case management. Thus, assessing RTW self-efficacy and RTW expectation may be of value early in the OR process, before a follow-up meeting at the job center or with an employer. Some sick-listed persons found the self-reporting approach meaningful, as some questions provided reflections on important aspects of their functioning and RTW process, and thereby a questionnaire could improve their involvement. The dilemma of “control over against support to clients” was raised by both sick-listed workers and professionals, revealing the uneven power relation, which must be acknowledged in self-reported assessment. Further, knowledge was lacking among sick-listed persons on the OR processes as well as among some professionals, particularly on which approaches are evidence-based in OR case management practice. The findings add knowledge to the complexity of case management and how important the inclusion of contextual factors is in work functioning assessment—yet to be systematized and aligned in Danish job centers.

## Data Availability

The datasets presented in this article are not readily available because of Municipals’ rules. Requests to access the datasets should be directed to the corresponding author.

## Appendix A

Preparation document for a rehabilitation plan. (The Danish Agency for Labour Market and Recruitment,. 2019).
