# Supplementary material for: Usability of self-reported assessment of work functioning in municipal occupational rehabilitation teams: A qualitative study
Source: Front Rehabil Sci. 2023 Jan 19;3:971574. doi: 10.3389/fresc.2022.971574 (PMC9893786; doi:10.3389/fresc.2022.971574)
Supplement: Supplementary file 1 [file Presentation1.pdf]

1.1 Borgerens oplysninger

Navn: ____________________

Cpr-nr : ____________________

Adresse: ____________________

1.2 Familie og netværk

Partner: ____________________

Børn: ____________________

1.3 Kontaktpersoner

Sagsbehandler: ____________________

Læge: ____________________

Andre kontaktpersoner : ____________________

____

____

____

____

1.4 Seneste job og uddannelse

Beskrivelse	Periode
Skriv seneste job her	
Skriv seneste uddannelse her	

1.5 Andre sager

Marker om der er andre sager.

Sag		Kontaktperson
Aktuel sag på børneområdet	Ja ○ Nej ○	
Aktuel sag på voksen- socialområdet	Ja ○ Nej ○	
Arbejdsskadesag eller ulykkesforsikringssag	Ja ○ Nej ○	

1.6 Seneste aktiviteter (praktikker og tilbud)

Aktivitet	Formål og udbytte	Periode

tilføj række fjern rækken

1.7 Seneste forsørgelse

Forsørgelse	Periode

tilføj række fjern rækken

1.8 Helbredsoplysninger

Attest	Afsender (fx praktiserende læge)	Dato for modtagelse

tilføj række fjern rækken

1.9 Bilag

Bilag	Afsender (fx arbejdsgiver)	Dato for modtagelse

tilføj række fjern rækken

2.1 Personlig beskrivelse

2.2 Erfaring (erhvervserfaring, praktikker og frivilligt arbejde)

Rolle: ______________________________ Sted: ____________________________________ Periode: _____________________________

Beskriv dine funktioner og arbejdsopgaver:

I hvor høj grad var du glad for forløbet?

○ I høj grad ○ I nogen grad ○ Hverken/eller ○ I mindre grad ○ I lav grad

Uddyb hvad der var godt eller mindre godt ved forløbet:

Rolle: ______________________________ Sted: ____________________________________ Periode: _____________________________

Beskriv dine funktioner og arbejdsopgaver:

I hvor høj grad var du glad for forløbet?

○ I høj grad ○ I nogen grad ○ Hverken/eller ○ I mindre grad ○ I lav grad

Uddyb hvad der var godt eller mindre godt ved forløbet:

tilføj række fjern rækken

2.3 Uddannelse, kurser og beviser

Tilføj her dine uddannelser, fx 9. klasse eller student. Tilføj også andre kurser eller beviser som fx kørekort eller hygiejnebevis.

Niveau / Type	Beskrivelse	Periode

tilføj række fjern rækken

2.4 Fritid

Her kan du beskrive, hvad du laver på en god dag, hvor du ikke har andre forpligtelser eller gøremål. Det kan være en dag, hvor du selv bestemmer, hvad du vil bruge tiden på

2.5 Hverdag

Her kan du beskrive dine hverdagsrutiner og hjemlige opgaver. Du kan også beskrive en typisk dag i dit liv fra morgen til aften.

2.6 Evner

Her kan du tilføje de evner, du har brugt i de roller, du har haft i arbejde, praktik eller frivilligt arbejde. Tilføj også de evner, du bruger i dine fritidsaktiviteter og din hverdag.

Dine styrker er de evner, som du er glad for at bruge og som giver dig energi. Beskriv dine evner og angiv, hvor glad du er for at bruge dem på en skala fra 1 til 4, hvor 1 er mindst glad og 4 er mest glad.

Beskrivelse	Hvor glad er du for at bruge denne evne?	Angiv på en skala fra 1 til 4
		① ② ③ ④
		① ② ③ ④
		① ② ③ ④
		① ② ③ ④

tilføj række   fjern rækken

3.1 Evner

Spørgsmål 1: Tror du, at dine erfaringer og evner kan bruges på en arbejdsplads?

- 1) Jeg er sikker på, jeg har noget at tilbyde en arbejdsplads
- 2) Jeg er for det meste sikker på, jeg har noget at tilbyde en arbejdsplads
- 3) Det svinger meget. Jeg er indimellem usikker på, om jeg har noget at tilbyde en arbejdsplads
- 4) Jeg tvivler meget på, jeg har noget at tilbyde en arbejdsplads
- 5) Jeg tror ikke, jeg har noget at tilbyde en arbejdsplads

Spørgsmål 2: Tror du, at du kan varetage et arbejde (på fuld tid, deltid eller få timer)?

- 1) Jeg er sikker på, at jeg kan varetage et arbejde
- 2) Jeg er nogenlunde sikker på, at jeg kan varetage et arbejde
- 3) Jeg er usikker på, om jeg kan varetage et arbejde
- 4) Jeg tvivler meget på, at jeg kan varetage et arbejde
- 5) Jeg tror slet ikke, at jeg kan varetage et arbejde

Spørgsmål kun til unge under 30 år uden en erhvervskompetencegivende uddannelse. Tror du, at du på et tidspunkt vil være i stand til at gennemføre en uddannelse?

- 1) Jeg er sikker på, at jeg på et tidspunkt vil være i stand til at gennemføre en uddannelse
- 2) Jeg er nogenlunde sikker på, at jeg på et tidspunkt vil være i stand til at gennemføre en uddannelse
- 3) Jeg er usikker på, om jeg på et tidspunkt vil være i stand til at gennemføre en uddannelse
- 4) Jeg tvivler meget på, at jeg på et tidspunkt vil være i stand til at gennemføre en uddannelse
- 5) Jeg tror slet ikke, at jeg på et tidspunkt vil være i stand til at gennemføre en uddannelse

3.2 Netværk

Spørgsmål 3: Hvor god er du til at samarbejde med andre?

- ◯ 1) Jeg har rigtig nemt ved at samarbejde med andre
- ◯ 2) Jeg har for det meste nemt ved at samarbejde med andre
- ◯ 3) Det svinger. Nogle gange er jeg god til det, andre gange ikke
- ◯ 4) Jeg har tit svært ved at samarbejde med andre
- ◯ 5) Jeg har rigtig svært ved at samarbejde med andre

Spørgsmål 4: Har du støtte fra familie og venner?

- ◯ 1) Min familie/venner støtter mig meget
- ◯ 2) Min familie/venner støtter mig for det meste
- ◯ 3) Det svinger. Nogle gange støtter familie/venner, andre gange ikke
- ◯ 4) Min familie/venner støtter mig ikke særlig tit, når jeg har behov for det
- ◯ 5) Min familie/venner støtter mig næsten aldrig, når jeg har brug for det

3.3 Helbred

Spørgsmål 5: Hvordan vil du alt i alt vurdere dit (fysiske og psykiske) helbred i forhold til at kunne varetage et arbejde (på fuld tid, deltid eller få timer)?

- ○ 1) Mit helbred er ikke i vejen for, at jeg kan arbejde
- ○ 2) Mit helbred er ikke i vejen for, at jeg kan arbejde, men kan sætte enkelte begrænsninger
- ○ 3) Det svinger. Indimellem gør mit helbred det svært at arbejde
- ○ 4) Jeg vil måske kunne varetage et job på få timer
- ○ 5) Mit helbred sætter store begrænsninger for, at jeg kan arbejde

Spørgsmål 6: Har du overskud i hverdagen til at fokusere på at få et arbejde, herunder virksomhedspraktik eller et uddannelsesforløb?

- ○ 1) Jeg kan sagtens overskue at fokusere på det
- ○ 2) Jeg kan for det meste overskue at fokusere på det
- ○ 3) Det svinger. Nogle gange kan jeg overskue det, andre gange ikke
- ○ 4) Jeg kan for det meste ikke overskue at fokusere på det
- ○ 5) Jeg kan næsten aldrig overskue at fokusere på det

3.4 Mål

Spørgsmål 7: Har du en idé om hvilket arbejde, du gerne vil have?

- ○ 1) Jeg ved lige hvilket arbejde, jeg gerne vil have
- ○ 2) Jeg har en idé om, hvilke typer arbejde jeg gerne vil have
- ○ 3) Det svinger. Nogle gange har jeg en klar idé, andre gange ikke
- ○ 4) Jeg har få, men uklare idéer om, hvilke typer arbejde jeg gerne vil have
- ○ 5) Jeg aner ikke, hvilke typer arbejde jeg gerne vil have

Spørgsmål 8: Ved du, hvad du skal gøre for at forbedre dine muligheder for at få et arbejde (på fuld tid, deltid eller få timer)?

- ○ 1) Jeg ved helt klart, hvad jeg skal gøre for at nærme mig et arbejde
- ○ 2) Jeg ved en del om, hvad jeg skal gøre for at nærme mig et arbejde
- ○ 3) Jeg ved nogenlunde, hvordan jeg kan nærme mig et arbejde, men er også noget i tvivl
- ○ 4) Jeg ved kun lidt om, hvordan jeg kan nærme mig et arbejde
- ○ 5) Jeg ved ikke, hvordan jeg kan nærme mig et arbejde

4.1 Borgerens bemærkninger

Hvis du har yderligere bemærkninger, kan du skrive dem her:

4.2 Sagsbehandlers bemærkninger

Lås dokumentet

OBS! Dokumentet kan ikke redigeres, efter det er blevet låst.
